# Is Cortisol Excretion Independent of Menstrual Cycle Day? A Longitudinal Evaluation of First Morning Urinary Specimens

**DOI:** 10.1371/journal.pone.0018242

**Published:** 2011-03-31

**Authors:** Pablo A. Nepomnaschy, Rachel M. Altman, Rita Watterson, Caroll Co, Daniel S. McConnell, Barry G. England

**Affiliations:** 1 Faculty of Health Sciences, Simon Fraser University, Burnaby, British Columbia, Canada; 2 Department of Statistics and Actuarial Science, Simon Fraser University, Burnaby, British Columbia, Canada; 3 School of Public Health, University of Michigan, Ann Arbor, Michigan, United States of America; 4 Department of Pathology, University of Michigan, Ann Arbor, Michigan, United States of America; University of Illinois at Champaign-Urbana, United States of America

## Abstract

**Background:**

Cortisol is frequently used as a marker of physiologic stress levels. Using cortisol for that purpose, however, requires a thorough understanding of its normal longitudinal variability. The current understanding of longitudinal variability of basal cortisol secretion in women is very limited. It is often assumed, for example, that basal cortisol profiles do not vary across the menstrual cycle. This is a critical assumption: if cortisol were to follow a time dependent pattern during the menstrual cycle, then ignoring this cyclic variation could lead to erroneous imputation of physiologic stress. Yet, the assumption that basal cortisol levels are stable across the menstrual cycle rests on partial and contradictory evidence. Here we conduct a thorough test of that assumption using data collected for up to a year from 25 women living in rural Guatemala.

**Methodology:**

We apply a linear mixed model to describe longitudinal first morning urinary cortisol profiles, accounting for differences in both mean and standard deviation of cortisol among women. To that aim we evaluate the fit of two alternative models. The first model assumes that cortisol does not vary with menstrual cycle day. The second assumes that cortisol mean varies across the menstrual cycle. Menstrual cycles are aligned on ovulation day (day 0). Follicular days are assigned negative numbers and luteal days positive numbers. When we compared Models 1 and 2 restricting our analysis to days between −14 (follicular) and day 14 (luteal) then day of the menstrual cycle did not emerge as a predictor of urinary cortisol levels (p-value >0.05). Yet, when we extended our analyses beyond that central 28-day-period then day of the menstrual cycle become a statistically significant predictor of cortisol levels.

**Significance:**

The observed trend suggests that studies including cycling women should account for day dependent variation in cortisol in cycles with long follicular and luteal phases.

## Introduction

Stress has been described as one of the most significant health problems in the 21^st^ century [1]. The physiologic response to stress is mediated by the hypothalamic-pituitary-adrenal axis (HPAA). HPAA function is linked to critical metabolic tasks such as immune response, cardiovascular function, reproductive physiology, and general well-being [2]–[5]. Understanding the basic function of this axis is, therefore, of critical importance both to monitor physiologic stress levels and to understand the pathways that link stress with negative health outcomes.

Cortisol is one of the most important end products of HPAA activation [6]. Energetic, health and psychosocial challenges lead to increases in this glucocorticoid's levels [7]–[9] and is, thus, frequently used to evaluate physiologic stress levels within and between individuals. When stimulated by endogenous and exogenous challenges, the paraventricular nucleus of the hypothalamus increases its production of corticotropin-releasing hormone (CRH), which in turn promotes the release of adrenocorticotropin (ACTH) by the anterior pituitary, leading to an increase in the secretion of glucocorticoids, including cortisol, by the adrenal cortex. Increases in cortisol levels trigger gluconeogenesis, resulting in higher levels of circulating glucose. Glucose provides energy to the tissues involved in responding to the challenges that trigger the activation of the HPAA in the first place [3]. Thus, cortisol levels are frequently used to monitor HPAA function and activation, and are interpreted as proxies of physiologic stress levels [10]–[12].

Yet, since the secretion of cortisol is affected by numerous factors its use as a marker of physiologic stress is not simple and should always be accompanied by proper controls [13]–[17]. One of these factors is the sex of an individual. In both sexes the neuroendocrine axes regulating stress response and reproductive function (the hypothalamic-pituitary-gonadal axis or HPGA) are intimately interconnected [18]–[20]. In women, however, the HPGA continuously transitions across reproductive stages and these changes appear to be associated with changes in HPAA functioning. Late pregnancy, for example, appears to be accompanied by hypercortisolemia [21] and the early post-partum period is characterized by changes in cortisol baseline levels and stress responsivity [22]–[25]. Thus, it is obviously important to consider women's reproductive status when assessing variations in HPAA function and physiologic stress levels. Nonetheless, our understanding of HPAA function is quite incomplete as we still lack a proper characterization of the changes in HPAA functioning across most reproductive transitions.

Longitudinal changes in HPAA functioning across women's menstrual cycles, for example, are yet to be properly characterized. It is often assumed that stress responsivity varies across the menstrual cycle but that baseline cortisol does not [26]. This is a critical assumption as it plays a vital role in the development of study designs of research focused on stress physiology and statistical analysis of the resulting data [6], [27]. If basal cortisol does not vary across the menstrual cycle then studies assessing HPAA functioning or stress physiology would not need to control for day of the menstrual cycle. In that case, individual basal cortisol levels could be simply assessed by collecting a small number of random specimens at any time during the menstrual cycle.

Despite the importance of this assumption to research on stress, however, the evidence suggesting that baseline cortisol levels do not vary across the menstrual cycle is scarce and contradictory. Most previous studies analyzing baseline cortisol levels across menstrual cycles have been conducted using cross-sectional designs, have been focused on particular days or narrow time windows within the cycle, and have been based mainly on two matrices with important limitations: blood and saliva. The ideal method with which to assess longitudinal variation in basal cortisol secretion across the menstrual cycle is to follow individual women across several menstrual cycles collecting bio-specimens as frequently as possible. Blood and saliva may not be the most appropriate matrices for these types of studies. Circulating levels of cortisol change rapidly. Thus, blood and saliva concentrations of this glucocorticoid are affected by instantaneous HPAA responses to any of a broad variety of ephemorous challenges. Some participants may, for example, experience an increase in cortisol levels triggered by the anticipatory anxiety generated by the impending prick that precedes blood collection. Additionally, blood collection is invasive, uncomfortable and carries a risk of infection, which makes it a poor choice for long-term studies that require repetitive sampling. The collection of saliva is less invasive and comparatively easier than that of blood. In fact, saliva has been successfully used in combination with experimental stress challenges to assess stress reactivity in different phases of the menstrual cycle [18], [28], [29]. Yet its use in non-experimental settings is complicated by the numerous non-stress related ephemorous factors that can affect cortisol levels, including normal physical activity and the consumption of food, caffeine, or alcohol [13]–[17]. Thus, despite its advantages for the experimental evaluation of stress reactivity, saliva has clear limitations as a matrix with which to assess basal cortisol profiles longitudinally in natural settings.

First morning urine, on the other hand, provides an integrated measure of overnight cortisol secretion, a time period that is less likely to be affected by the ephemorous, mostly diurnal, confounders mentioned above. Furthermore, as urine can be self-collected and collection is relatively non-invasive, it is a matrix that lends itself to be used in designs that involve repetitive sampling across long time periods.

Here we present longitudinal analyses of cortisol levels in first morning urinary specimens provided by Kakchiquel women from rural Guatemala. These analyses contribute to our understanding of basal HPAA functioning across women's reproductive transitions and provide critical information on the use of a matrix that lends itself to naturalistic longitudinal studies while, simultaneously, reducing the effect of relatively ephemorous confounding factors. Our results will be useful in informing study designs and protocols involving cortisol as a marker of HPAA function and evaluating physiologic stress in women across the menstrual cycle.

## Results

**Figure 1:**
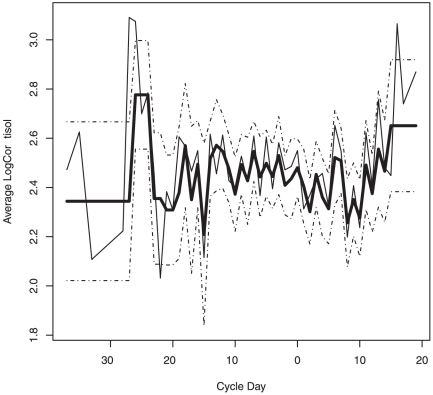
Observed and fitted cortisol profiles using Model 2_i_. The thick black line represents the fitted means for Model 2_i_ and the dotted lines represent the standard errors for the individual estimated means. The thin black line represents the observed average cortisol levels for each menstrual cycle day.

**Figure 2:**
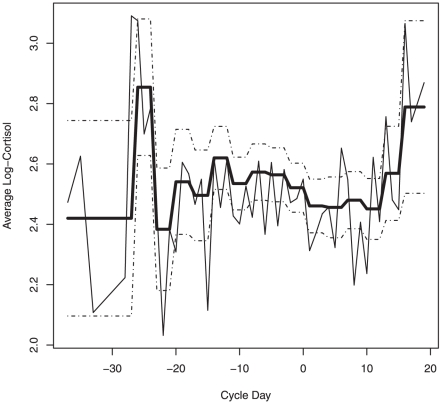
Observed and fitted cortisol profiles using Model 2_ii_. The thick black line represents the fitted means for Model 2_ii_ and the dotted lines represent the standard errors for the individual estimated means. The thin black line represents the observed average cortisol levels for each menstrual cycle day.

We first fit the proposed models only to the data collected during the 28 days traditionally considered to be the length of standard menstrual cycles (days −14≤ ovulation ≤14). When only those data were included in the analyses we found no evidence that either Model 2_i_ nor Model 2_ii_ fit better than Model 1 (p-values = 0.098 and 0.161, respectively). In other words, our results provide no evidence that cortisol level varied by day of the menstrual cycle during cycle days −14≤t≤14. However, when we used the entire data set (including the data from follicular and luteal phases that lasted more than 14 days), we found evidence that Model 2_i_ and Model 2_ii_ fit better than Model 1 (p-values = 0.041 and 0.009, respectively). These results imply that in cycles with long follicular and luteal phases (>14 days) cortisol levels vary with the day of the menstrual cycle. Figures 1 and 2 illustrate the fitted mean cortisol levels based on Model 2_i_ and Model 2_ii_, respectively. These figures suggest that the observed variation in overnight excretion of cortisol levels may be explained by differences characterizing the onset and last days of long menstrual cycles. Our sample size, however, did not permit the formal investigation of which days or set of days were significantly different in terms of mean cortisol levels.

## Discussion

To our knowledge, this is the first longitudinal description of first morning urinary cortisol levels across the full menstrual cycle in humans. Our analyses suggest that day of the menstrual cycle is a significant predictor of first morning urinary cortisol levels in cycles with follicular or luteal phases lasting longer than 14 days. Our sample size does not allow us to determine which specific days during the menstrual cycle differ in terms of cortisol secretion. However, the lack of significant differences in cortisol means when we restrict our analyses to the 14 days immediately preceding and following ovulation suggest that the differences in cortisol levels occur beyond the central 28 day period.

Animal studies suggest that sex steroids could affect basal HPAA functioning [30]. Female rats exhibit higher glucocorticoid levels (corticosterone) during proestrus, when estradiol is higher, than during estrous [31]–[33]. Furthermore, experimental ovariectomy leads to a fall in corticosterone, which is resolved via the administration of exogenous estradiol [34], [35]. Yet the association observed between sexual steroids and HPAA functioning in rats may not be directly extrapolable to the human case. While there is evidence to suggest that ovarian function affects stress response in women [18], [28], [36], basal cortisol levels are commonly assumed not to vary across the menstrual cycle [26]. This assumption, however, rests on limited and contradictory evidence.

Results from previous human studies evaluating variation in basal cortisol levels across menstrual cycle range broadly. Some studies find no differences between menstrual cycle phases, while others report higher cortisol levels in either the follicular or the luteal phase or within phase changes in basal cortisol levels. Most of these studies use blood or saliva as their matrix. Symonds and colleagues, for example, report no significant differences in cortisol levels assessed in salivary specimens collected one day at mid-follicular and one day at mid-luteal [37]. Similarly, Kudielka and Kirschbaum [6] report no effect of menstrual cycle phase on cortisol in salivary samples taken directly after awakening as well as 15, 30, 45, and 60 minutes thereafter. In one of the few studies based on daily blood samples, Saxena and colleagues [38] evaluated cortisol levels across the menstrual cycle in 6 healthy women. The blood specimens collected between 7 am and 8 am between days −11 to +11 of the menstrual cycles showed no cyclic variations in cortisol levels. These and other studies with similar results [39]–[41] have led to the common assumption that there is no variation in basal cortisol levels across the menstrual cycle.

In contrast, other researchers using the same matrices do report variations in cortisol levels across the menstrual cycle. Genazzani and colleagues [42] collected blood specimens from 5 women experiencing “regular” menstrual cycles. Their protocol asked that blood be drawn at 7∶30 am after a light breakfast between days −11 and +14 of the menstrual cycle. They report significant differences within the follicular phase with cortisol lower between days −7 and −4 and higher on day −2. Beck and colleagues [43] evaluated adrenocortical function 10 and 24 days after the last menstrual period and also report cortisol in plasma to be significantly higher in the sample collected during the follicular phase.

The lack of consistency in results is likely a consequence of the wide variability in designs and methods across studies, combined with relatively small sample sizes and the limitations of the matrices used. For example, sampling schedules and protocols can have a significant impact on the level of within and between individuals' variability in cortisol levels when using blood or saliva. Cortisol secretion follows circadian patterns and can be affected by the participants' wake up time, the time elapsed between wake-up time and the collection of the sample, and the events that took place in that interval. To reduce the influence of these confounders, early studies based on blood specimens scheduled specimens' collection very early in the morning and mostly before participants had breakfast (e.g. [40], [42], [43]). However, controlling for the stimuli that women are exposed to between that time and the moment they arrive at the laboratory to get their blood drawn is extremely difficult. Furthermore, the prospect of having blood extracted can act, for some participants, as a stressor itself. All of these factors introduce variability in the data obtained, reducing statistical power and, with it, the ability of researchers to detect changes in basal adrenocortical cortisol secretion across the menstrual cycle. Some teams have attempted to solve these issues by working with saliva which, as it can be self-collected, can be obtained immediately after women wake up each morning (e.g. [6], [37]). Loose adherence to the sampling schedule by participants, however, leads to the same problems of differential exposure to stimuli [6], [27]. Another important issue is the period of the menstrual cycle evaluated. Many of the previous studies have been based on single or, in some cases, a few samples collected at specific days during each menstrual cycle phase. Our results and those of Genazzani and colleagues [42] suggest, however, that there may be a significant amount of variation in cortisol secretion within each phase. Thus, cortisol levels assessed on specific days may not represent mean phase levels and do not provide information about within phase variability or the longitudinal profile of cortisol secretion across each phase. A related problem is that day of the menstrual cycle is frequently imputed by counting days since the onset of the last menstrual bleeding (e.g. [6], [37], [44]). This method, however, is quite inaccurate in terms of identifying the timing of the biologic processes that may ultimately affect cortisol secretion, such as the stage of follicular development or ovulation [45]. Inaccuracies in the identification of key biologic events during the menstrual cycle introduce yet another source of variability, increasing the risk of committing a type II statistical error (i.e., failing to detect differences in cortisol production between menstrual cycle phases).

Our study design helped to reduce the effect of several of the problems described above. We used a longitudinal approach including complete menstrual cycles (in most cases more than one per participant) and first morning urine to assess both cortisol and reproductive hormones (LH, FSH, E_1_C and PdG) to impute menstrual cycle day using a key reproductive event (day of ovulation). This methodology presumably increased our statistical power, aiding in the detection of variation in overnight adrenal production of cortisol in menstrual cycles with prolonged inter-ovulatory-intervals (IOIs). The nature of our data precludes us from exploring the proximate function (if any) of the observed variations in cortisol level. The observed variation in overnight cortisol level may either be directly involved in the physiologic pathways leading to the prolongation of the IOI period or be a consequence of said prolongation. Long IOIs can result from prolonged follicular and/or luteal phases. HPAA activation can lead to the prolongation of the follicular phase [46], [47]. Thus, the observed variability in cortisol levels in the early days of cycles of long follicular phases could actually be the cause of the follicular extension. On the other hand, HPAA activation is unlikely to explain prolonged luteal phases. HPAA activation has been linked to poor luteal function [47], [48], which would result in an early shedding of the endometrium and consequently shorter rather than longer luteal phases. It could also be argued that the observed variation in cortisol excretion may be linked to the implantation of conceptuses that failed to produce detectable levels of human chorionic gonadotrophin (hCG). Such conceptuses, however, would fail to rescue the corpus luteum and, consequently, are unlikely to lead to prolonged luteal phases.

Alternatively, the increased overnight cortisol excretion in long IOIs may be explained by reproductive and immune processes that take place during the transition between consecutive menstrual cycles. Cortisol variability could be associated with an exacerbation of inflammatory processes that accompany either the multiple follicular waves that take place between ovulatory events [49]–[51] or menstruation [52]–[54]. Previous studies provide evidence that glucocorticoids play an important modulating role in those inflammatory processes [52]–[54].

Our finding that, in long cycles, cortisol depends on day of menstrual cycle has important implications in terms of study design and statistical analysis. For example, to obtain an unbiased estimate of a woman's typical cortisol level, cortisol measurements must be taken throughout her menstrual cycle, namely, both during the central 28 day window around ovulation, and on each day outside of this window. Moreover, if cortisol is used as the response variable in a regression model that does not account for day of menstrual cycle, the estimates of the effects of the predictors that do appear in the model will be biased. Thus, studies using cortisol as a marker of physiologic stress levels in cycling women should not disregard as a potential confounder the day of the menstrual cycle in which a given sample has been collected. Rather, statistical comparison of cortisol levels within and between women should either be based on similar menstrual cycle days or adjust for day of the menstrual cycle.

In sum, our results suggest that basal overnight cortisol secretion vary across the menstrual cycle. First morning urine presents clear advantages over blood and saliva as a matrix with which to evaluate long-term longitudinal variations in basal cortisol levels across women's reproductive transitions. Our sample was collected from a rural indigenous population with high ethnic homogeneity and comparably less variability than other populations in terms of physical schedules and energy intake. While small differences in terms of absolute mean cortisol levels may exist among ethnic groups, adrenal function and general secretion profiles are expected to be universal across all women. It would however be important to replicate our study in communities with higher levels of ethnic heterogeneity and a broader range of energetic balances. Larger sample sizes will be required to determine the specific mathematical function describing mean cortisol levels across the menstrual cycle. Our results, nonetheless, suggest that studies involving the measurement of cortisol levels in cycling women should account for day dependent variation in cortisol, particularly in cycles with long follicular and luteal phases.

## Materials and Methods

### Study population and criteria for participant inclusion

This paper is based on data collected in the context of the Society, Environment and Reproduction (SER) study. Fieldwork took place over 12 months between the years 2000 and 2001 in a rural Kaqchikel Mayan community located in the southwest highlands of Guatemala. This community was composed at the time of 1,159 inhabitants who were almost exclusively Kaqchikel Mayan. All women within this population who fit the following profile were invited to participate: living with a co-resident male partner, not pregnant, not using any form of chemical contraceptive method, had given birth at least once in the past, and whose last birth had taken place at least 6 months prior to the onset of the study. Additional details on the characteristics of the population have been reported elsewhere [48], [55]. During the first half of the study recruitment was restricted to women aged 18–32 years, but later the upper age limit was expanded to 40 years to increase the sample size.

### The sample

Throughout the year, 61 women (about three-quarters of those eligible) volunteered to participate. Twenty-five (25) of the 61 participants cycled at least once during the study and experienced a total of 84 menstrual cycles (mean  = 3.4, SD = 3.05, median  = 2). Of these 84 menstrual cycles, 29 cycles had long follicular phases (>14 days), 5 had long luteal phases (> days) and one cycle had both long follicular and luteal phases. This summary of cycle length data is, however, affected by the large number of “censored” cycles contained in our sample (41 out of 84). The onset and end of each woman's participation in our study were unlikely to coincide with the first or last day of a cycle and, thus, were unlikely to be full cycles. The analyses performed for this paper are based on the data corresponding to the women who had resumed ovarian function after their last birth. The ages of these 25 women ranged from 18–39 years but, because of the original design, the age distribution was heavily weighted toward the mid-20s (mean  = 25.4 years, SD = 5.3 years, median  = 25 years).

This research was approved by the Research Ethics Board of Simon Fraser University. As most individuals in the study population were illiterate, informed consent from the participants was obtained orally. The consent document was read in Kakchiquel Mayan by a female research assistant (a native Kakchiquel speaker) and signed by each volunteer with a cross, finger print or name initials, according to her preference.

### Data and specimen collection

Data and urine specimen collection were performed by trained local female field assistants. Every other day, for a total of three times each week, assistants visited participants in their homes and gathered first morning urine samples. Following standard urine collection protocols, urine specimens were collected by each participant in clean, dry, nonreactive plastic containers that we provided the night before. Samples were kept on ice until assistants returned to the laboratory (<2 hours from the urinary void). Two-ml aliquots from the original specimens were stored frozen at −10°C in the field. Samples were shipped on dry ice to the **CLASS** laboratory at the University of Michigan, where they were stored at −80°C until analysis.

### Hormonal assays

Concentrations of urinary free cortisol, estrone conjugates (E_1_C), pregnandiol glucuronide (PdG), luteinizing hormone (LH), follicle stimulating hormone (FSH), and hCG were determined using immunoassays developed in CLASS laboratory for use with the Bayer Automated Chemiluminescence System (ACS-180) immunoassay analyzer. Creatinine was assayed using a spectrophotometric assay. All samples from a single participant were run on the same assay and in duplicate. Outliers were identified and the samples rerun. Ranges and intra- and inter-assay coefficients of variation (IACV and IECV, respectively) were within acceptable ranges. Creatinine: range  = 0.05–1.4 mg/ml, IACV  = 5.4% IECV  = 9.8%; cortisol: range  = 0.2–75 mg/dl, IACV  = 2.0%, IECV  = 6.5%; E_1_C: range  = 5.10–408.0 ng/ml, IACV  = 3.8%, IECV  = 6.5%; PdG: range  = 0.005–25.5 mg/ml, IACV  = 3.6%, IECV  = 11.6%; LH: range  = 0.1–53.1 mIU/ml, IACV  = 3.5%, IECV  = 5.4%; FSH: range  = 0.3–144.0 mIU/ml, IACV  = 2.3%, IECV  = 5.8% [48].

### Data analysis

#### Characterization of menstrual cycles and cycle day attribution

To control for urine dilution we divided the concentration of each hormone by the concentration of creatinine in the same sample. We then log transformed the creatinine corrected hormonal measurements, after which the data became approximately normally distributed. Log transformed creatinine corrected hormone levels were used for all our statistical analyses and thus, from this point forward, are simply referred to as cortisol.

Menstrual cycles were considered to begin on the first day of vaginal bleeding and end the day before the next bleeding. If reports of vaginal bleeding were missing or confusing we imputed last day of the cycle to the day in which PdG levels fell to 40% of its luteal peak and remained low for ≥2days [48]. Cycles presenting a 3-fold rise in PdG levels above baseline were considered ovulatory [56]. The time of ovulation was inferred using an algorithm based on the urinary ratio of E_1_C/PdG [57] and verified using the presence of LH and FSH surges. Menstrual cycles were aligned to the estimated day of ovulation, which was designated “day 0.” Follicular days were given negative numbers and luteal days positive numbers.

#### Confounding factors

Cortisol secretion can be affected by circadian rhythms, physical activity, food consumption, smoking, caffeine, alcohol, and steroid medications [13]–[17]. First morning specimens provide a proxy for overnight cortisol excretion. Working with overnight cortisol excretion minimizes the effects that circadian rhythms have on this metabolite's profile. None of the participants smoked or consumed alcohol. Urine samples were collected as soon as the participants woke up each morning and before they consumed food or performed any major physical activity, eliminating the influence of those confounders. Our sample of women was relatively homogeneous in terms of age. Thus, we did not evaluate the possible impact of age on cortisol levels in our models. See also Nepomnaschy *et al.* (2004) [48].

### Statistical analysis

We compared two linear mixed models for cortisol. Mixed models take into account both fixed (e.g., day of the menstrual cycle) and random (individual participant) effects. The random effect captures the effects of unmeasured variables that might explain some of the differences in cortisol among women, thus controlling for possible correlations among cortisol measurements from the same woman.

**Model 1** assumed that there was no variation in mean cortisol across the menstrual cycle. Specifically, we assumed:
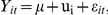
where 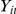 is the cortisol measurement on woman *i* on day *t*, 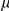 is the overall mean cortisol level, 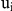 is the effect on the overall mean for woman *i*, and 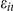 is the random error for woman *i* on day *t*. Furthermore, we assumed that:


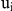
 is drawn from a normal distribution with mean 0 and variance 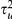



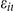
 is drawn from a normal distribution with mean 0 and variance 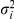



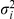
 is drawn from a gamma distribution with parameters common across women.

This model assumes that the mean cortisol level (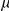) is flat across the menstrual cycle, but that this mean may differ among women (i.e., the mean level for woman *i* is 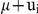). The random variable 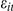 captures random error in the cortisol values (including increases due to stressful events). Data were plotted longitudinally by participant. Visual inspection of the resulting menstrual cycles profiles suggest that the amount by which cortisol fluctuates around the mean differs across women. Some women, in particular, have nearly identical measurements over time, whereas others have highly variable measurements. For this reason, we allow the variance of 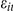 to vary among women (according to a gamma distribution).

**Model 2** is similar to Model 1, but in this case mean cortisol is allowed to vary as a function of time across the menstrual cycle. In particular, we let
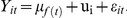



We evaluated two versions of this last model. In version 2_i_ of this model cortisol means were estimated for each day between days −19 and 14, inclusive. Outside this period we had very few observations for some days (<8 observations for each day). Thus, to avoid problems with parameter estimation we defined the following time windows: day< = −27, −27<day< = −24, −24<day< = −22, −22<day< = −20, day>14. We allowed mean cortisol level to vary among time windows, but assumed that the mean was constant within each window. See Figure 1.

For version 2_ii_ we defined 3-day windows across the menstrual cycle except at the extremes of the distribution of follicular and luteal days where we grouped days into two windows: “very early follicular days” (days ≤−27) and “very late luteal days” (days >15). Again, we allowed mean cortisol to vary among these windows but assumed that it remained constant within each window. Model 2_ii_ has fewer parameters than Model 2_i_, possibly providing more power to detect differences among cortisol means across the menstrual cycle. The disadvantage of Model 2_ii_ is that it assumes that cortisol profiles are flat over each 3 day window. See Figure 2.

No other formal analyses of cycle day effect were conducted. However, before deciding that our proposed longitudinal model provided a reasonable description of the data, we explored alternative models. Specifically, we fitted a standard linear mixed model (i.e. with common variability of cortisol across women), but the model allowing differences in cortisol variability across women seemed to fit better and was therefore preferred over the standard model. We also used (informal) graphical methods for two purposes. First, we looked for, but did not find, an obvious autocorrelation structure describing the cortisol measurements on individual women (a finding consistent with the assumptions of our final model). Second, we checked whether other variables such as age and a recent previous pregnancy loss could explain cortisol patterns or outliers but found no evidence of such associations.
